# DNA methylation markers for oral cancer detection in non- and minimally invasive samples: a systematic review

**DOI:** 10.1186/s13148-024-01716-9

**Published:** 2024-08-13

**Authors:** Óscar Rapado-González, Sofia Salta, Rafael López-López, Rui Henrique, María Mercedes Suárez-Cunqueiro, Carmen Jerónimo

**Affiliations:** 1 Department of Surgery and Medical-Surgical Specialties, Medicine and Dentistry School, Universidade de Santiago de Compostela (USC), 15782 Santiago de Compostela, Spain; 2 Galician Precision Oncology Research Group (ONCOGAL), Medicine and Dentistry School, Universidade de Santiago de Compostela (USC), 15782 Santiago de Compostela, Spain; 3 Liquid Biopsy Analysis Unit, Translational Medical Oncology Group (ONCOMET), Health Research Institute of Santiago (IDIS), 15706 Santiago de Compostela, Spain; 4 Centro de Investigación Biomédica en Red en Cáncer (CIBERONC), Instituto de Salud Carlos III, 28029 Madrid, Spain; 5 Cancer Biology & Epigenetics Group, Research Center of IPO Porto (CI-IPOP), Portuguese Oncology Institute of Porto (IPO Porto) / Porto Comprehensive Cancer Center - Raquel Seruca (Porto.CCC) & CI-IPOP@RISE (Health Research Network), Rua Dr. António Bernardino de Almeida, 4200-072 Porto, Portugal; 6 Translational Medical Oncology Group (ONCOMET), Health Research Institute of Santiago (IDIS), Complexo Hospitalario Universitario de Santiago de Compostela (CHUS, SERGAS), 15706 Santiago de Compostela, Spain; 7 Department of Pathology, Portuguese Oncology Institute of Porto (IPO Porto) / Porto Comprehensive Cancer Center - Raquel Seruca (Porto.CCC) & CI-IPOP@RISE (Health Research Network), Rua Dr. António Bernardino de Almeida, 4200-072 Porto, Portugal; 8 Department of Pathology and Molecular Immunology, ICBAS-School of Medicine and Biomedical Sciences, University of Porto, Rua Jorge Viterbo Ferreira 228, 4050-313 Porto, Portugal

**Keywords:** DNA methylation, Oral cancer, Diagnosis, Screening, Epigenetics, Liquid biopsies

## Abstract

More than 50% of oral cancer (OC) patients are diagnosed with advanced-stage disease associated with poor prognosis and quality of life, supporting an urgent need to improve early OC detection. The identification of effective molecular markers by minimally invasive approaches has emerged as a promising strategy for OC screening. This systematic review summarizes and evaluates the performance of the DNA methylation markers identified in non- or minimally invasive samples for OC detection. PubMed’s MEDLINE, Scopus, Embase, and Cochrane Library databases were systematically searched for studies that evaluated DNA methylation markers in non-invasive and/or minimally invasive samples (oral rinse/saliva, oral brush, and blood) from OC patients. Two investigators independently extracted data on study population characteristics, candidate methylation markers, testing samples, DNA methylation assay, and performance diagnostic outcomes. Methodological study quality was assessed with the Quality Assessment for Studies of Diagnostic Accuracy-2 tool. Thirty-one studies met the inclusion criteria for this systematic review. DNA methylation markers were evaluated in oral rinse/saliva (*n* = 17), oral brush (*n* = 9), and blood (*n* = 7) samples. Methylation-specific PCR (MSP) and quantitative-MSP were the most common DNA methylation assays. Regarding diagnostic performance values for salivary, oral brush, and blood DNA methylation markers, sensitivity and specificity ranged between 3.4–100% and 21–100%, 9–100% and 26.8–100%, 22–70% and 45.45–100%, respectively. Different gene methylation panels showed good diagnostic performance for OC detection. This systematic review discloses the promising value of testing DNA methylation markers in non-invasive (saliva or oral rinse) or minimally invasive (oral brush or blood) samples as a novel strategy for OC detection. However, further validation in large, multicenter, and prospective study cohorts must be carried out to confirm the clinical value of specific DNA methylation markers in this setting.

**Supplementary Information:**

The online version contains supplementary material available at 10.1186/s13148-024-01716-9.

## Introduction

Oral cancer (OC) represents a global public health problem with an estimated 377,713 new cases and 177,757 deaths in 2020 [1]. Due to the lack of specific symptoms at the early stage and delays in the diagnosis, more than 50% of OC patients are diagnosed with advanced-stage disease entailing invasive therapies associated with several disabilities that compromise patient quality of life [2]. Unfortunately, more than half of the patients diagnosed with advanced clinical stage suffer relapse in the course of the disease, with a 5-year overall survival rate below 50% [3]. Conversely, this survival rate increases up to 90% when OC is diagnosed at early stage [4] where treatment outcomes are more effective, supporting an urgent need to improve early OC detection.

Screening by visual oral examination is the standard initial step for the detection of oral potentially malignant disorders (OPMDs) and early-stage OC lesions, requiring a scalpel biopsy and subsequent histopathological analysis for diagnosing oral lesions [5, 6]. Despite the easy access of the oral cavity to examination, screening by visual oral examination has low sensitivity (66.7%) emphasizing the challenge that represents for clinicians the detection of OC at early stages and the identification of OPMD disorders in high-risk populations based on visual appearance only [7]. Various adjunctive tools to visual oral examination based on vital staining, cytology, or light-based detection have been applied for facilitating the recognition of OC and pre-cancerous lesions, but the low specificity of these methods has limited their application for OC screening in primary care [8].

In the last decade, research efforts have focused on the identification of effective molecular markers enabling cancer screening using minimally invasive approaches [9–11]. Particularly, aberrant DNA methylation has emerged as a promising tumor marker since this epigenetic alteration is an early event in carcinogenesis [12]. Thus, CpG island hypermethylation within gene promoter regions has been identified in oral carcinogenesis resulting in transcriptional silencing of several tumor suppressor genes involved in a broad range of cellular processes including cell cycle control, apoptosis, Wnt signaling, cell–cell adhesion, and DNA repair [13]. In this context, several studies have shown the potential of detecting DNA methylation markers in exfoliated oral cells obtained by oral brushing and/or oral saline rinsing for OC diagnosis [14–19]. In addition, since DNA methylation can be detected in different body fluids [20], various authors have explored the feasibility of testing DNA methylation biomarkers in liquid biopsies based on saliva and blood for head and neck cancer detection [21, 22]. In this vein, a few genome-wide DNA methylation profiling studies have been performed in liquid biopsies from OC patients using microarray and sequencing-based DNA methylation technologies [23–26]. Thus, Viet et al. identified a diagnostic classifier based on 41 gene loci from 34 genes by comparing pre- and postoperative saliva samples from OC patients using Illumina GoldenGate Methylation [26]. Later, Langevin et al. interrogated the DNA methylation profile in oral rinses using the Infinium HumanMethylation450 BeadArray and they identified a methylation classifier comprising 22 CpG islands for predicting oral and pharyngeal cancers [23]. Recently, Patel et al. discovered multiple differential methylated regions (DMR) for discriminating pre- and post-treatment plasma samples by cell-free DNA methylation profiling using methyl-CpG binding protein sequencing [24]. Similarly, Adeoye et al. characterized the salivary methylome profile by reduced representation bisulfite sequencing (RRBS), identifying by machine learning a model based on 11 DMR with high diagnostic accuracy for OC detection [25]. Hence, these data support the usefulness of genome-wide DNA methylation strategies for discovering novel methylation biomarkers with potential diagnostic and prognostic value for OC management. However, a comprehensive and critical overview of the DNA methylation markers identified in exfoliated oral cells and liquid biopsies in OC is lacking.

In this systematic review, we summarize and evaluate the performance of DNA methylation markers tested in non-invasive or minimally invasive samples for OC detection to provide evidence regarding their potential clinical value for OC screening.

## Material and methods

### Protocol and registration

This systematic review was carried out according to the Preferred Reported Items for Systematic Reviews and Meta-Analysis (PRISMA) guidelines [27] and was registered in the PROSPERO database at the Centre of Reviews and Dissemination, University of York, UK, with the registration number CRD42023487606.

### Search strategy

PubMed’s MEDLINE, Scopus, Embase, and Cochrane Library databases were systematically searched for eligible articles until October 2023. The search strategy for the four databases is detailed in Additional file 1. Two reviewers (ORG and SS) independently screened the title and abstract of all identified articles as part of the first selection round. Then, full texts of selected articles were retrieved and reviewed for eligibility criteria. Disagreements regarding  eligibility were resolved through discussion with a third reviewer (MMSC). Additionally, reference lists of relevant studies and reviews were reviewed to identify relevant articles. The articles identified by means of different database searches were managed using RefWorks software (https://www.refworks.com/content/path_learn/faqs.asp), accessed November 9, 2023), and duplicate items were removed using the associated tools.

### Eligibility criteria

Articles met the inclusion criteria if they were case–control or cohort studies that evaluated the diagnostic performance of DNA methylation markers (without limitation of methylation assay type) in non-invasive and/or minimally invasive samples (blood, saliva, oral rinse, and oral brush) comparing OC patients with controls (healthy individuals, OPMD patients or benign oral conditions). Articles were excluded if they were (1) reviews, editorial letters, case reports, or conference abstracts; (2) duplicate publications; (3) written in non-English language; or (4) analyzing DNA methylation on animal models or cell lines.

### Data collection and extraction

Two investigators (ORG and SS) independently extracted data using a standardized form of each eligible study. Any disagreements between reviewers were resolved by consensus. Data collection included first author, publication year, study population country, number of cases and controls, tumor characteristics (tumor anatomic location/TNM stage), type of control group (healthy/benign/OPMD), type of sample (blood/saliva/oral rinse/oral brush), specific methylation markers, DNA methylation assay, and performance outcomes in detecting oral cancer. If the information was incomplete, attempts were made to contact the authors to request the missing information.

### Quality assessment

The methodological quality of the included studies was separately assessed by two independent investigators (ORG and SS) using the Quality Assessment for Studies of Diagnostic Accuracy-2 (QUADAS-2) tool [28], as recommended by the Healthcare Research and Quality Agency, Cochrane Collaboration, and the U.K. National Institute for Health and Clinical Excellence. The QUADAS-2 tool is designed to assess the risk of bias and applicability of primary diagnostic accuracy studies across four domains: patient selection, index test, reference test, flow, and timing. All domains are evaluated in terms of risk of bias, and only the first three domains are also assessed in terms of concerns regarding the applicability of the findings to the review question. Discrepancies between the two investigators were resolved by a third reviewer (MMSC).

## Results

### Literature overview

**Fig. 1:**
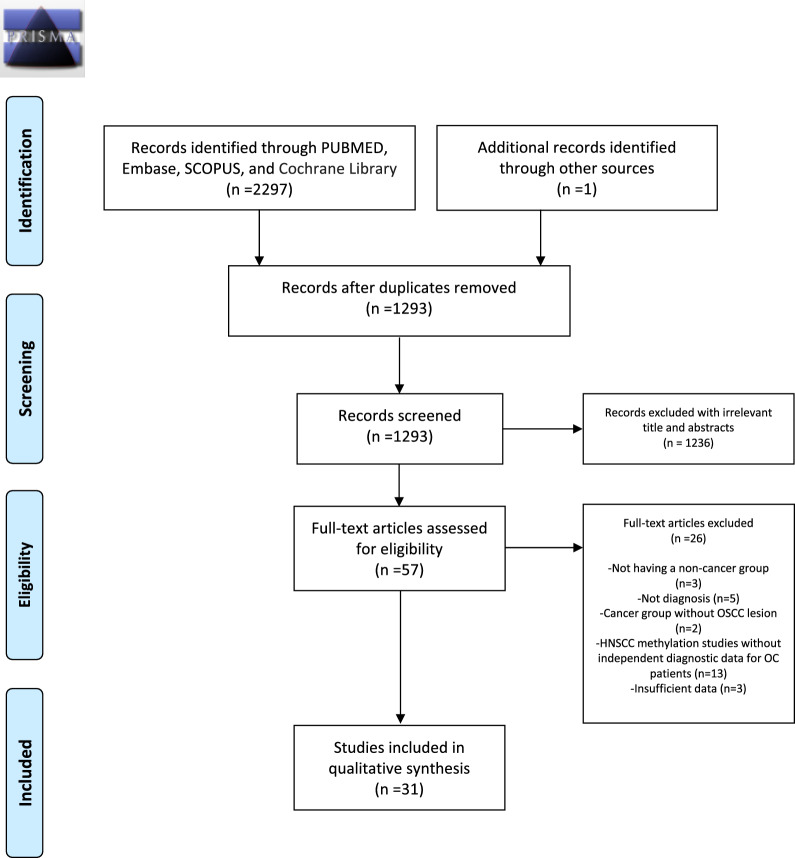
PRISMA flow diagram

A total of 1293 articles were screened after removing duplicates through search in different electronic databases. After screening title and abstract review, 57 articles were found eligible for full-text-assessment. Of these, 26 articles were excluded for different reasons including: not having a non-cancer comparator group (*n* = 3); study outcomes not related to diagnosis (*n* = 5); inclusion of treated OC patients as cancer group (*n* = 2); research conducted in head and neck squamous cell carcinoma without independent results for oral tumors (*n* = 13); or not having sufficient diagnostic data (*n* = 3). The literature search and study selection process for this systematic review are shown in Fig. 1 by a PRISMA flowchart.

### Study characteristics

A total of 31 articles published between 2001 and 2022 investigated different DNA methylation markers [14–19, 26, 29–52]. Based on the study design, 29 case–control and three cohort studies were included. A healthy control group was identified in 27 studies with sample sizes ranging from 3 to 200. One of these studies also considered the postoperative OC group as a control [26]. In addition, 10 studies also included OPMD groups [14, 15, 17, 29–35]. In two studies [14, 15], the OPMDs (homogeneous thin leukoplakia, homogenous thick leukoplakia, non-homogeneous leukoplakia, erythroleukoplakia, and verrucous hyperplasia) were classified according to their histopathological diagnosis (hyperplasia/hyperkeratosis, mild dysplasia, moderate dysplasia, and severe dysplasia) for DNA methylation analysis while, in another study [29], oral lesions were characterized into high- or low-grade intraepithelial lesions and oral lichen planus. Only three studies [32, 36, 37] included a benign control group (oral fibroma and inflammatory hyperplasia). Seventeen studies were conducted in Asian populations, including five studies from Japan, three from China, three from Taiwan, three from India, two from Thailand, and one from Sri Lanka. In addition, 10 studies were conducted in American populations, including six studies from the USA, two from Brazil, one from Mexico, and one from Colombia, and four studies were conducted in Italy. The studies included DNA methylation markers assessed in oral rinse (*n* = 11), oral brush (*n* = 9), blood (*n* = 7), and saliva (*n* = 4) samples. In two studies [32, 36, 37], oral rinse was combined with an oral brush to increase the number of exfoliated cells from the oral cavity. Regarding the DNA methylation assay, methylation-specific polymerase chain reaction (MSP) and quantitative-MSP (qMSP) were used in 17 and 9 studies, respectively. Other studies used targeted next-generation sequencing (NGS) (*n* = 3), methylation arrays (*n* = 1), and ferrocenyl-napthalene diimide-based electrochemical hybridization assay (*n* = 1). Most of the studies evaluated the methylation status of specific genes previously reported methylated in the literature [14–16, 19, 30–34, 36, 38–46] while other studies performed a gene methylation profiling [26, 47] or applied a bioinformatic approach to public methylation microarray data for identifying target genes [17, 18].

### Diagnostic performance of salivary DNA methylation markers for OC detection

A total of 17 articles evaluated the promoter methylation status of different genes in saliva samples, including oral rinse (*n* = 10), whole saliva (*n* = 4), and oral rinse combined with oral brush (*n* = 3) (Table 1). Methylation of six (*CDKN2A*, *DAPK1*, *MGMT*, *TIMP3*, *NID2*, *EDNRB,* and *RASSF1*) out of the 62 genes was reported ≥ 2 times, and that of the remaining genes was reported only once. In five studies [14, 36, 38, 46, 47], the performance measurements of methylated genes were reported both individually and in different combinations while in 15 studies the sensitivity and specificity values were individually reported or calculated from available data only for specific genes. In addition, two studies reported only the diagnostic performance of gene panels [19, 26].

**Table 1:**
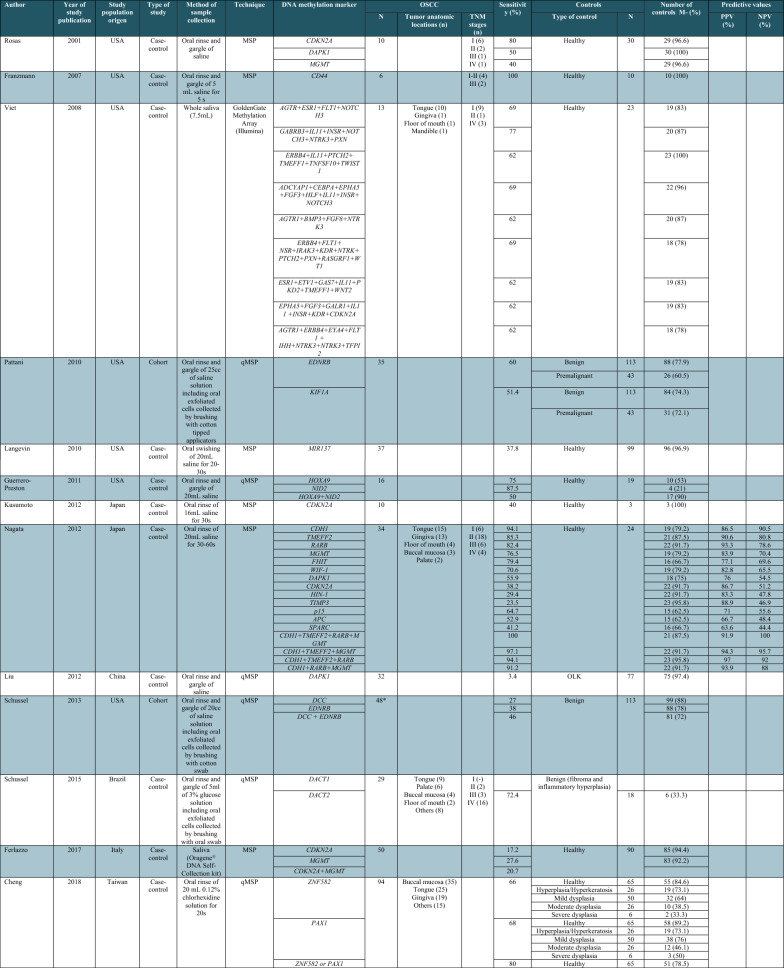

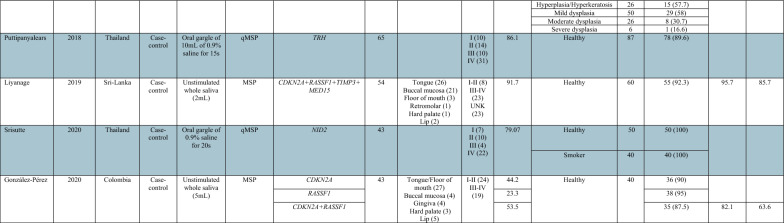
Descriptive characteristics of DNA methylation studies using saliva and/or oral rinse samples for oral cancer diagnosis

OSCC, oral squamous cell carcinoma; OLK, oral leukoplakia; MSP, methylation-specific PCR; qMSP, quantitative methylation-specific PCR. *Epithelial dysplasia/cancer

Overall, sensitivity for OC detection using salivary DNA methylation markers ranged from 3.4 to 100% with specificity varying from 21 to 100%. Single genes with sensitivities ≥ 75% for OC detection included *CDH1* [46], *TMEFF2* [46], *RARB* [46], *MGMT* [46], *FHIT* [46], *HOXA9* [23], *NID2* [17], *TRH* [44], *CDKN2A* [39], and *CD44* [41]. Among these, *NID2*, *TRH*, *CDKN2A*, *MGMT* and *CD44* showed specificity ≥ 90%. Various gene combinations showed good diagnostic performance for OC detection. Nagata et al. reported 4 different methylation panels consisting of the combination of *ECAD*, *TMEFF2*, *RARB*, and *MGMT* with sensitivity ranging between 91.2 and 100%, specificity ranging between 87.5 and 95.8%, positive predictive value (PPV) ranging between 91.9 and 97%, and negative predictive value (NPV) ranging between 88 and 100% [46]. Liyanage et al. also identified a methylation panel comprising *CDKN2A*, *RASSF1*, *TIMP3*, and *MED15* disclosing 91.7% sensitivity, 92.3% specificity, 95.7% PPV and 85.7% NPV. They further evaluated the clinical performance of this 4-gene methylation panel by tenfold cross-validation, resulting in 83.3% sensitivity and 92.3% specificity for identifying OC cases [19]. Regarding the potential of salivary DNA methylation markers for detecting OPMDs, Cheng et al. evaluated the sensitivity of methylated *ZNF582* and *PAX1* for detecting OPMDs with different grades of dysplasia, including hyperplasia (27% and 27%), mild dysplasia (36% and 24%), moderate dysplasia (62% and 54%), and severe dysplasia (67% and 50%), respectively. Interestingly, the combination of both genes disclosed sensitivity values ranging from 42% for hyperplasia/hyperkeratosis to 83% for severe dysplasia lesions [14].

### Diagnostic performance of oral brush DNA methylation markers for OC detection

Nine articles evaluated the methylation status of 27 genes in oral brush samples (Table 2). Single genes with sensitivity ≥ 75% for OC detection comprised *PAX1* [15, 48], *SOX1* [48], *ZNF582* [15], *NID2* [17], *MLH1* [40], *ZAP70* [29], *GP1BB* [29], and *TERT* [31]. Among these, *PAX1* [15], *ZNF582* [15], *NID2* [17], *MLH1* [40], *ZAP70* [29], and *GP1BB* [29] displayed specificity ≥ 90%. Three studies [15, 16, 30] evaluated the diagnostic performance of gene panels. In the Cheng et al. study, methylation of the *ZNF582*/*PAX1* gene panel allowed the detection of moderate dysplasia or worse oral lesions with 93% sensitivity and 65% specificity [15]. Morandi et al. reported a 13-gene methylation panel with 96.5% sensitivity and 100% specificity for differentiating OC from healthy controls. Further validation in an independent cohort confirmed the high diagnostic accuracy of this gene methylation panel [30]. Recently, the same research group evaluated the potential diagnostic value of this 13-gene methylation panel in an Italian multicenter study, reporting a sensitivity of 93.6%, a specificity of 84.9%, a PPV of 86.6%, and a NPV of 92.8% [16]. Regarding the potential of DNA methylation markers for detecting OPMDs by oral brush testing [15, 17, 29–31], the sensitivity values ranged from 28.6 to 100%. In Cheng et al. study, the sensitivity for OPMD detection based on *ZNF582* and *PAX1* gene methylation was reported for different grades of oral dysplasia, including hyperplasia (27% and 12%), mild dysplasia (68% and 32%), and moderate dysplasia (87% and 56%), respectively. The gene methylation panel based on *ZNF582* and *PAX1* yielded a sensitivity ranging from 31% for hyperplasia/hyperkeratosis to 90% for moderate dysplasia oral lesions [15]. In another study, Morandi et al. reported a 13-gene methylation panel with 100% sensitivity for the detection of high-grade squamous intraepithelial lesions [30].

**Table 2:**
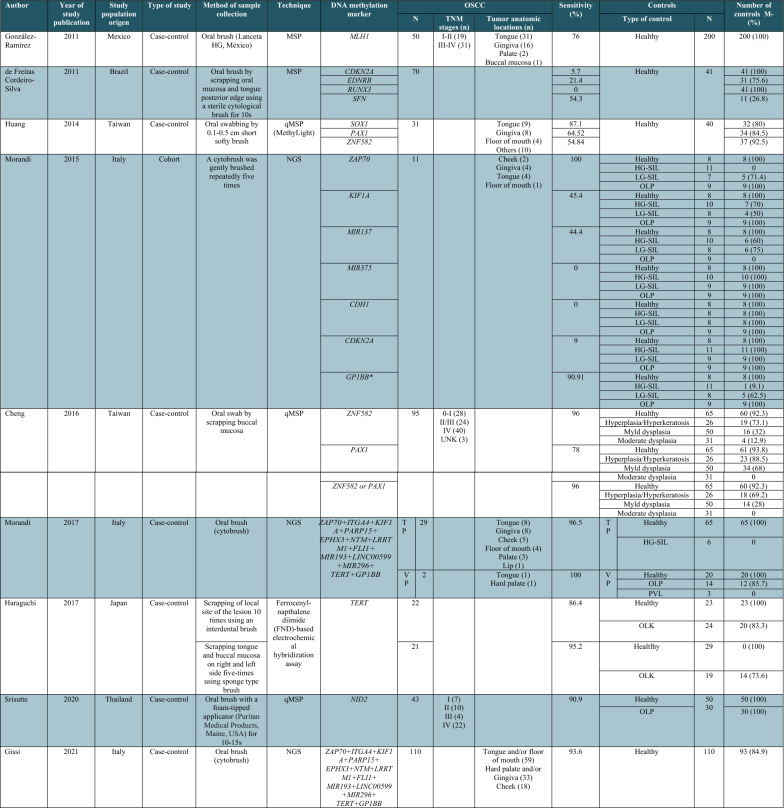
Descriptive characteristics of DNA methylation studies using oral brush samples for oral cancer diagnosis

HG-SIL, high-grade squamous intraepithelial lesion; LG-SIL, low-grade squamous intraepithelial lesion; OLP, oral lichen planus; OLK, oral leukoplakia; PVL, proliferative verrucous leukoplakia;; MSP, methylation-specific PCR; qMSP, quantitative methylation-specific PCR; TP, training dataset; VP, validation dataset; UNK, unknown

*Hypomethylation

### Diagnostic performance of blood DNA methylation markers for OC detection

Seven articles evaluated the methylation status of 6 genes (*CDKN2A*, *Cdh13*, *MGMT*, *COX2*, *CDH1*, *DAPK1*, *LATS1,* and *LATS2*) in blood samples (Table 3). Two studies [43, 44] assessed gene methylation in serum samples and five studies in blood. Overall, sensitivity for OC detection based on blood DNA methylation markers ranged from 22 to 70% with specificity ranging from 45.4 to 100%. Concerning the potential of DNA methylation markers for detecting OPMDs, the sensitivity values ranged from 18.1 to 54.5%. In Bhatia et al. study, the sensitivity for OPMD detection based on *MGMT* and *CDKN2A* methylation was calculated for different OPMDs, including leukoplakia (41% and 45%), oral leukoplakia without dysplasia (55% and 18%), oral submucous fibrosis (31% and 46%), and oral lichen planus (25% and 0%), respectively [34]. Liu et al. reported 20.9% sensitivity for oral lichen planus detection based on *DAPK1* methylation [33] while in Xu et al. study the sensitivity values for oral submucous fibrosis based on *COX2* and *CDH1* methylation were 30% and 52%, respectively [35].

**Table 3:**
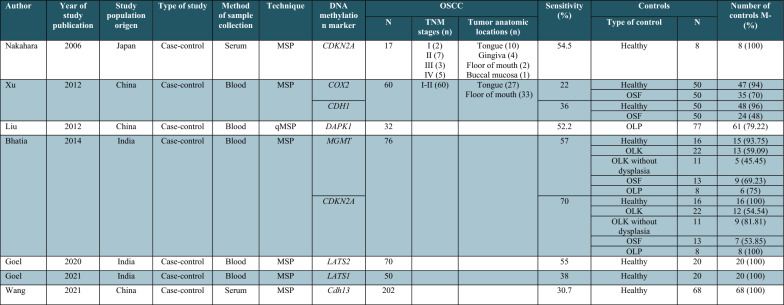
Descriptive characteristics of DNA methylation studies using blood samples for oral cancer diagnosis

OSCC, oral squamous cell carcinoma; OLP, oral lichen planus; OLK, oral leukoplakia; OSF, oral submucous fibrosis; MSP, methylation-specific PCR; qMSP, quantitative methylation- specific PCR

### Quality assessment

The results of the quality assessment of individual studies using the QUADAS-2 tool are shown in Additional Table S1 and Additional Fig. S1. In terms of risk of bias, most included studies disclosed high patient selection bias which was related to a case–control study design and the lack of information regarding random or consecutive patient enrollment. Only five studies [29, 30, 43, 45, 49] explicitly described consecutive recruitment of patients with clinical and histological OC diagnosis. Regarding the index test domain, all methylation tests (index test) were interpreted with the knowledge of the reference standard (tissue biopsy). Oral cancer and precancerous lesions received the same reference standard that allows to confirm of the disease diagnosis by histopathological examination. Moreover, data regarding the time interval between sample collection for index testing and the application of the reference standard were not provided clearly in the study’s methodology. As for applicability concerns, all domains (patient selection, index test, and reference standard) were considered to have an overall low risk of bias.

## Discussion

To the best of our knowledge, this systematic review is the first to provide an overview of DNA methylation markers assessed in non- and minimally invasive samples from OC and OPMDs. In addition, the performance of the single-gene methylation markers and methylation panels for OC and OPMD detection is reported.

To date, tissue biopsy of the suspicious lesion followed by histopathological assessment remains the gold standard for OPMDs and OC diagnosis. Nonetheless, this procedure cannot be applicable for population screening and has important drawbacks such as invasiveness, sampling bias, patient discomfort and requires trained health professionals, justifying the need for developing minimally invasive diagnostic procedures. In this vein, DNA methylation identified in non- and minimally invasive samples has emerged as an attractive tool for early cancer detection. Oral brushes and salivary rinses were the samples most used for testing DNA methylation markers among the studies included in this systematic review. Salivary rinse collection was mainly obtained by rinsing and/or gargling the oral cavity with normal saline while a few studies used other solutions based on glucose or chlorhexidine [15, 36]. Regarding oral brushing, oral swab or cytobrush applicators were used for harvesting oral cells [15, 17, 30, 48]. Interestingly, in two studies [32, 36], salivary rinses were enriched in exfoliated oral cells collected by brushing the surface of the oral cavity to obtain a higher representation of cells located deeper in the epithelium. From the point of view of a screening program, saliva is an attractive diagnostic sample due to its non-invasive and easy collection, not requiring trained personnel and special equipment for sampling, making it very cost-effective [53]. Previously, our research group assessed the overall performance of salivary DNA hypermethylation for head and neck cancer detection, disclosing a pooled sensitivity and specificity of 39% and 87%, respectively. Interestingly, subgroup analysis by tumor anatomic location revealed higher sensitivity in oral and oropharyngeal tumors, supporting the potential clinical value of saliva for identifying and testing specific methylated markers related to oral carcinogenesis [54]. Recently, Adeoye et al. evaluated the efficacy of testing individual DNA methylation markers in saliva samples and oral swabs for OC diagnosis. They found similar sensitivity values (72% vs. 71.2%, respectively); however, the specificity was higher in oral swabs (97.1%) compared to oral rinses (88.4%) [55].

Among the studies included in our present review, two of them evaluated the methylation levels of specific genes in oral rinse and oral brush samples [14, 17]. Cheng et al. compared *ZNF582* and *PAX1* gene promoter methylation levels in both sample types showing sensitivity and specificity values slightly higher in oral scrapes compared to oral rinse samples [14]. In the same line, Srisutte et al. reported higher *NID2* methylation frequency in oral swab (90.9%) compared to oral rinse (77.3%) samples from OC patients [17]. These findings reflect the improvement in sensitivity when gene methylation is tested in oral brush. Nonetheless, this specific approach requires a specialist in oral pathology for collections and may cause bleeding and painful sensations.

Among the 31 studies evaluating the diagnostic potential of DNA methylation markers included in this systematic review, more than 50 different genes were reported methylated, either single or in gene panels, but only 12 genes (*CDKN2A*, *DAPK1*, *MGMT*, *TIMP3*, *RASSF1*, *CDH1*, *EDNRB*, *ZNF582*, *PAX1*, *NID2*, *KIF1A,* and *MIR137*) were reported methylated ≥ 2 times, which reduces the comparability of the findings for specific genes among studies. Furthermore, most investigations reported only the methylation of single genes while the diagnostic potential of combining different methylated genes was explored in only a small number of these studies [14, 16, 19, 30, 36, 46, 47]. In this vein, Nagata et al. reported four methylation panels based on the combination of *CDH1*, *TMEFF2*, *RARB,* and/or *MGMT*, disclosing sensitivity ≥ 91.2%, specificity ≥ 87.5%, PPV ≥ 91.9% and NPV ≥ 88% for differentiating OC patients from healthy individuals [46]. Likewise, Liyanage et al. evaluated *CDKN2A*, *RASSF1*, *TIMP3*, and *MED15* methylation in saliva from OC patients and healthy controls and found that the combination of these four methylated genes yielded high diagnostic accuracy for OC, with 91.7% sensitivity, 92.3% specificity, 95.7% PPV, and 85.7% NPV [19]. More recently, Gissi et al. evaluated the diagnostic performance of a 13-gene methylation panel in an Italian multicenter study, disclosing 93.6% sensitivity, 84.9% specificity, 86.6% PPV, and 92.8% NPV for differentiating OC from healthy controls using oral brush testing [16]. Overall, these data emphasize the improvement in diagnostic accuracy enabled by salivary methylation tests when various gene methylation markers are combined and tested in saliva. Although further validation in independent and multicenter large cohort studies is mandatory to confirm the sensitivity and specificity values, PPV and NPV must be also assessed to determine the diagnostic tests’ validity as screening tools.

With the purpose of developing non-invasive molecular screening strategies, research in this field performed over the last decade has shown the potential clinical value of different molecular combinations based on microRNAs [56], mRNA [57], proteins [58], metabolites [59] or gene somatic mutations [60] for OC detection. For instance, meta-analytic evidence on the diagnostic accuracy of blood and salivary miRNAs revealed a pooled sensitivity of 78% and specificity of 82% for OC detection [61], whereas for salivary mRNA markers a higher pooled sensitivity (91%) and specificity (90%) were reported [62]. In contrast, a recent meta-analysis revealed, for IL-8, a pooled sensitivity of 41% and specificity of 69%, and for IL-1β a pooled sensitivity and specificity of 26% and 47%, respectively, which reflects the limited diagnostic accuracy of these salivary cytokines for OC detection [62]. Comparing these results with those of salivary gene methylation panels, the latter display a superior diagnostic performance supporting its potential as clinically useful biomarkers for OC detection.

Since OC may be preceded by OPMD, various authors have explored the presence of DNA methylation in these oral lesions as an early molecular marker of oral carcinogenesis. Thus, Cheng et al. showed that *ZNF582* and *PAX1* methylation rates increased gradually with the severity of oral lesions (normal–hyperplasia/hyperkeratosis–mild dysplasia–moderate/severe dysplasia–squamous cell carcinoma) suggesting a key role for DNA methylation in neoplastic transformation. Interestingly, *ZNF582* methylation displayed 85% sensitivity and 87% specificity for discriminating mild dysplasia or worse oral lesions, whereas *PAX1* methylation showed 72% sensitivity and 86% specificity for discriminating moderate dysplasia or worse oral lesions, emphasizing the potential of both markers for the detection of oral dysplasia and OC [15]. In another study, *ZAP70* hypermethylation was reported in all cases of OC and high-grade squamous intraepithelial lesions, whereas no methylation was found in oral lichen planus lesions and healthy individuals, demonstrating a promising role of *ZAP70* methylation for early OC detection. Other hypermethylated genes include *KIF1A* and *MIR137* although sensitivity and specificity were lower [29]. Interestingly, Bhatia et al. also reported significant methylation of *MGMT* and *CDKN2A* genes in leukoplakia with dysplasia and OC patients, suggesting the possibility of detecting blood epigenetic alterations involved in the progression of premalignant oral condition to cancerous state [34]. These data demonstrate the presence of various tumor-specific epigenetic alterations in OPMDs, underlining the role of DNA methylation in oral carcinogenesis.

Among the reviewed studies, MSP was one of the most used methods for detecting DNA methylation markers, being used in 16 studies. Although MSP displays high sensitivity, its clinical applicability is challenging owing to its non-quantitative character, which may lead to an increase in false positive results and test variability due to assay conditions (e.g., primer design, annealing temperature, cycle number) [63]. Hence, the findings of these studies should be interpreted with caution. More recent studies have used quantitative methylation techniques such as qMSP [17, 18, 47, 48] or NGS [29, 30], which display high sensitivity and specificity for detecting tumor-specific DNA methylation alterations. Interestingly, most studies utilized a targeted methylation assay for investigating the methylation status of specific gene sequences, with only a few investigations making use of high-throughput technologies such as NGS and microarrays. In Morandi et al. study, the methylation status of 19 gene targets was characterized by bisulfite conversion of DNA followed by NGS [30], whereas Viet et al. used the GoldenGate methylation array assay to discover novel salivary methylation biomarkers for early OC detection [26]. Thus, ongoing advances in sequencing and microarray technology are very likely to decisively influence strategies to identify novel methylation markers for OC detection in future investigations.

Although this systematic review provides a comprehensive overview of all DNA methylation biomarkers investigated in non- and minimally invasive samples (oral brush, oral rinse/saliva, and blood) for detecting OC and OPMDs, it has some limitations. Indeed, a high risk of bias was identified in most of the included studies and those published in non-English language were excluded; thus, the risk of having missed some relevant studies exists. Moreover, comparability of the results was not possible due to variability in the methodology and the limited number of specific gene methylation studies which mostly did not evaluate the accuracy of selected markers concerning PPV and NPV values. Consequently, a meta-analysis evaluating the diagnostic potential of different DNA methylation markers could not be carried out.

## Conclusions

In summary, this systematic review demonstrates the potential of DNA methylation markers for OC detection using non-invasive or minimally invasive samples. Importantly, several DNA methylation markers have been identified as promising diagnostic markers, with very good or even excellent performance. Further validation in larger and prospective study cohorts must be carried out, however, to assess the real clinical value for early OC and OPMD detection.

### Supplementary Information

Supplementary Material 1: Additional File 1. Detailed search algorithms for electronic search strategy. Table S1. Quality assessment ratings using the QUADAS-2 Scale for reviewed studies (n=31). Figure S1. Bar graph of QUADAS-2 results of bias and applicability for reviewed studies (n=31).

## Data Availability

No datasets were generated or analyzed during the current study.

## Abbreviations

DMR: Differential methylated regions
MSP: Methylation-specific polymerase chain reaction
NGS: Next-generation sequencing
OC: Oral cancer
OPMD: Oral potentially malignant disorders
PRISMA: Preferred Reported Items for Systematic Reviews and Meta-Analysis
qMSP: Quantitative methylation-specific polymerase chain reaction
QUADAS-2: Quality Assessment for Studies of Diagnostic Accuracy-2
