# Supplementary material for: DNA methylation markers for oral cancer detection in non- and minimally invasive samples: a systematic review
Source: Clin Epigenetics. 2024 Aug 13;16:105. doi: 10.1186/s13148-024-01716-9 (PMC11323632; doi:10.1186/s13148-024-01716-9)

RAPADO-GONZALEZ et al-ADDITIONAL File- 1

**Additional File 1 —** Detailed search algorithms for electronic search strategy.

**Additional Table S1 —** Quality assessment ratings using the QUADAS-2 Scale for reviewed studies (n = 31). **Additional Fig. S1 —** Bar graph of QUADAS-2 results of bias and applicability for reviewed studies (n = 31).

**Additional File 1 —** Detailed search algorithms for electronic search strategy.

**PubMed**

(((“Squamous Cell Carcinoma of Head and Neck” [Mesh] OR “head and neck squamous cell carcinoma” [Title/Abstract] OR HNSCC [Title/Abstract] OR “oral squamous cell carcinoma” [Title/Abstract] OR “oral cavity squamous cell carcinoma” [Title/Abstract] OR “oral cancer” [Title/Abstract] OR OSCC [Title/Abstract]) AND (Methylation [Mesh] OR Methylat* [Title/Abstract] OR hypermethylat* [Title/Abstract] OR “promoter hypermethylation” [Title/Abstract] OR “promoter gene methylation” [Title/Abstract] OR epigenetic [Mesh]) AND (diagnosis [Mesh] OR diagnos*[ Title/Abstract] OR detection [Title/Abstract] OR "early detection of cancer" [Mesh] OR "early diagnosis" [Mesh] OR screening [Title/Abstract])

**OVID EMBASE**

(“Squamous Cell Carcinoma of Head and Neck” OR “head and neck squamous cell carcinoma” OR HNSCC OR “oral squamous cell carcinoma” OR “oral cavity squamous cell carcinoma” OR “oral cancer” OR OSCC) AND (Methylation OR Methylat* OR hypermethylat* OR “promoter hypermethylation” OR “promoter gene methylation” OR epigenetic) AND (diagnosis OR diagnos* OR detection OR "early detection of cancer" OR "early diagnosis" OR screening)

(("Squamous Cell Carcinoma of Head and Neck" OR "head and neck squamous cell carcinoma" OR HNSCC OR "oral squamous cell carcinoma" OR "oral cavity squamous cell carcinoma" OR "oral cancer" or OSCC) AND (Methylation or Methylat* OR hypermethylat* OR "promoter hypermethylation" OR "promoter gene methylation" OR epigenetic) AND (diagnosis OR diagnos* OR detection OR "early detection of cancer" OR "early diagnosis" OR screening)).mp. [mp=title, abstract, heading word, drug trade name, original title, device manufacturer, drug manufacturer, device trade name, keyword heading word, floating subheading word, candidate term word]

**SCOPUS**

TITLE-ABS-KEY (( "Squamous Cell Carcinoma of Head and Neck" OR "head and neck squamous cell carcinoma" OR hnscc OR "oral squamous cell carcinoma" OR "oral cavity squamous cell carcinoma" OR "oral cancer" OR oscc ) AND ( methylation OR methylat* OR hypermethylat* OR "promoter hypermethylation" OR "promoter gene methylation" OR epigenetic ) AND ( diagnosis OR diagnos* OR detection OR "early detection of cancer" OR "early diagnosis" OR screening ))

**Cochrane**

(“Squamous Cell Carcinoma of Head and Neck” OR “head and neck squamous cell carcinoma” OR HNSCC OR “oral squamous cell carcinoma” OR “oral cavity squamous cell carcinoma” OR “oral cancer” OR OSCC) AND (Methylation OR Methylat* OR hypermethylat* OR “promoter hypermethylation” OR “promoter gene methylation” OR epigenetic) AND (diagnosis OR diagnos* OR detection OR "early detection of cancer" OR "early diagnosis" OR screening):ti,ab,ky

**Additional Table S1 —** Quality assessment ratings using the QUADAS-2 Scale for reviewed studies (n = 31). N, no; Y, yes; U, unclear.

|  | **Patient Selection** | | | | | **Index Test** | | | | **Reference Standard** | | | | **Flow and Timing** | | | | |
| --- | --- | --- | --- | --- | --- | --- | --- | --- | --- | --- | --- | --- | --- | --- | --- | --- | --- | --- |
| **Signaling Questions** | **Q1** | **Q2** | **Q3** | **Risk of bias** | **Applicability concerns** | **Q4** | **Q5** | **Risk of bias** | **Applicability concerns** | **Q6** | **Q7** | **Risk of bias** | **Applicability concerns** | **Q8** | **Q9** | **Q10** | **Q11** | **Risk**  **of bias** |
| Rosas SLB, 2001 | N | N | Y | High | Low | N | N | High | Low | U | U | Unclear | Low | Y | U | U | Y | Unclear |
| Nakahara Y, 2006 | Y | N | Y | High | Low | N | N | High | Low | Y | Y | Low | Low | U | U | U | N | High |
| Franzmann EJ, 2007 | N | N | Y | High | Low | N | N | High | Low | Y | Y | Low | Low | N | Y | Y | N | High |
| Viet CT, 2008 | N | N | Y | High | Low | N | Y | High | Low | Y | Y | Low | Low | U | Y | Y | Y | Low |
| Pattani KM, 2010 | N | Y | Y | High | Low | N | Y | High | Low | Y | Y | Low | Low | U | Y | Y | Y | Unclear |
| Langevin SM, 2010 | Y | N | Y | High | Low | N | N | High | Low | Y | Y | Low | Low | N | U | U | Y | High |
| Guerrero-Preston R, 2011 | N | N | Y | High | Low | N | Y | High | Low | U | U | Unclear | Low | U | U | U | Y | Unclear |
| González-Ramírez I, 2011 | N | N | Y | High | Low | N | N | High | Low | Y | Y | Low | Low | Y | Y | Y | Y | Low |
| de Freitas Cordeiro-Silva MF, 2011 | N | N | Y | High | Low | N | N | High | Low | U | U | Unclear | Low | U | U | U | Y | Unclear |
| Nagata S, 2012 | N | N | Y | High | Low | N | Y | High | Low | U | U | Unclear | Low | N | U | U | Y | High |
| Ksumoto T, 2012 | Y | N | Y | High | Low | N | N | High | Low | U | U | Unclear | Low | N | U | U | Y | High |
| Liu Y, 2012 | N | N | Y | High | Low | N | Y | High | Low | Y | Y | Low | Low | Y | Y | Y | Y | Low |
| Xu C, 2012 | N | N | Y | High | Low | N | N | High | Low | Y | Y | Low | Low | N | Y | Y | Y | High |
| Schussel J, 2013 | N | Y | Y | High | Low | N | N | High | Low | Y | Y | Low | Low | Y | Y | Y | N | Low |
| Bathia V, 2014 | N | N | Y | High | Low | N | N | High | Low | U | U | Unclear | Low | U | U | U | Y | High |
| Huang YK, 2014 | N | N | Y | High | Low | N | Y | High | Low | Y | Y | Low | Low | N | Y | Y | Y | High |
| Schussel JL, 2015 | N | N | Y | High | Low | N | N | High | Low | Y | Y | Low | Low | N | Y | U | Y | High |
| Morandi L, 2015 | Y | N | Y | High | Low | N | N | High | Low | Y | Y | Low | Low | Y | Y | Y | Y | Low |
| Cheng SJ, 2016 | N | N | Y | High | Low | N | Y | High | Low | Y | Y | Low | Low | Y | Y | Y | N | Low |
| Morandi L, 2017 | Y | N | Y | High | Low | N | Y | High | Low | Y | Y | Low | Low | Y | Y | Y | Y | Low |
| Cheng SJ, 2018 | N | N | Y | High | Low | N | Y | High | Low | Y | Y | Low | Low | Y | Y | Y | N | Low |
| Ferlazzo N, 2017 | N | N | Y | High | Low | N | N | High | Low | U | U | Unclear | Low | U | U | U | Y | High |
| Haraguchi K, 2017 | N | N | Y | High | Low | N | Y | High | Low | U | U | Unclear | Low | N | U | U | Y | High |
| Puttipanyalears C, 2018 | Y | N | Y | High | Low | N | Y | High | Low | Y | Y | Low | Low | N | Y | Y | Y | High |
| Liyanage C, 2019 | N | N | Y | High | Low | N | Y | High | Low | Y | Y | Low | Low | U | Y | Y | Y | Low |
| Srisutte R, 2020 | N | N | Y | High | Low | N | N | High | Low | Y | Y | Low | Low | N | Y | Y | Y | High |
| Goel H, 2020 | N | N | Y | High | Low | N | N | High | Low | U | U | Unclear | Low | N | U | U | Y | High |
| Gissi DB, 2020 | N | N | Y | High | Low | N | Y | High | Low | Y | Y | Low | Low | Y | Y | Y | Y | Low |
| González-Pérez LV, 2020 | N | N | Y | High | Low | N | N | High | Low | Y | Y | Low | Low | N | U | U | N | High |
| Wang Q, 2021 | N | N | Y | High | Low | N | N | High | Low | Y | Y | Low | Low | Y | Y | Y | Y | Low |
| Goel H, 2021 | N | N | Y | High | Low | N | N | High | Low | U | U | Unclear | Low | N | U | U | Y | High |

**Additional Fig. S1 —** Bar graph of QUADAS-2 results of bias and applicability for reviewed studies (n = 31).


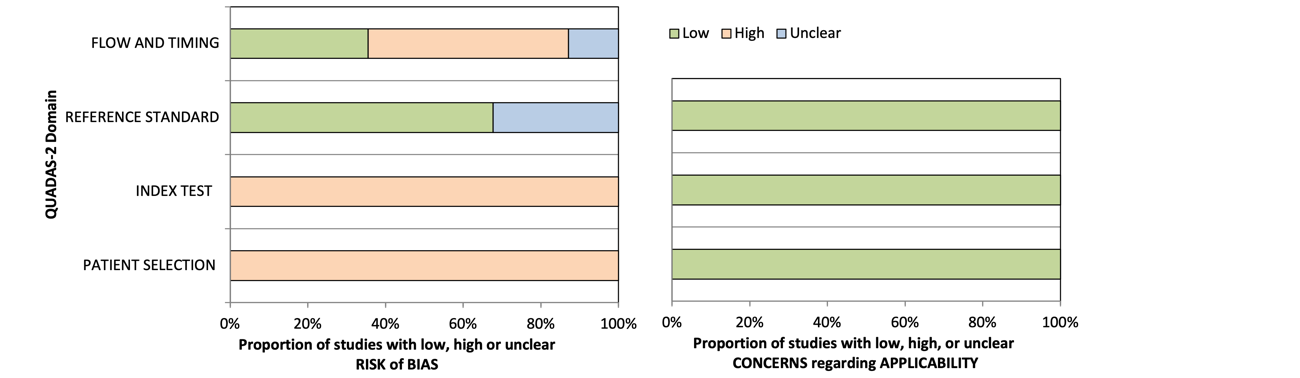

Supplement: Supplementary file 1 — Supplementary Material 1: Additional File 1. Detailed search algorithms for electronic search strategy. Table S1. Quality assessment ratings using the QUADAS-2 Scale for reviewed studies (n=31). Figure S1. Bar graph of QUADAS-2 results of bias and applicability for reviewed studies (n=31). [file 13148_2024_1716_MOESM1_ESM.docx]
